# Group processes and interoperability: A longitudinal case study analysis of the UK's civil contingency response to Covid‐19

**DOI:** 10.1111/1468-5973.12424

**Published:** 2022-07-07

**Authors:** Matthew Radburn, Clifford Stott, Rebecca Bryant, Bethan Morgan, Deborah Tallent, Louise Davidson

**Affiliations:** ^1^ School of Psychology Keele University Keele Staffordshire UK; ^2^ Staffordshire Fire and Rescue Service Stone Staffordshire UK; ^3^ Staffordshire Civil Contingencies Unit Stone Staffordshire UK; ^4^ Behavioural Science Team, Emergency Response Department Science & Technology, Health Protection Directorate Public Health England London UK; ^5^ School of Psychology University of Sussex Brighton UK

**Keywords:** civil contingency response, COVID‐19, emergency management, group processes, intergroup relations, Interoperability, social identity, strategic decision‐making

## Abstract

Our case study explored a Local Resilience Forum's (LRF) civil contingency response to COVID‐19 in the United Kingdom. We undertook 19 semistructured ethnographic longitudinal interviews, between March 25, 2020 and February 17, 2021, with a Director of a Civil Contingencies Unit and a Chief Fire Officer who both played key roles within their LRF. Within these interviews, we focused on their strategic level decision‐making and how their relationship with national government impacted on local processes and outcomes. Using a form of grounded theory, our data describe the chronological evolution of an increasingly effective localized approach toward outbreak control and a growing resilience in dealing with concurrent emergency incidents. However, we also highlight how national government organizations imposed central control on aspects of the response in ways that undermined or misaligned with local preparedness. Thus, during emergencies, central governments can undermine the principle of subsidiarity and damage the ways in which LRFs can help scaffold local resilience. Our work contributes to the theoretical understanding of the social psychological factors that can shape the behaviour of responder agencies during a prolonged crisis. In particular, the implications of our analysis for advancing our conceptual understanding of strategic decision‐making during emergencies are discussed.

## 1 INTRODUCTION

The United Kingdom's civil contingency response to Covid‐19 has involved a vast network of national and local response agencies. Therefore, a central issue has been interoperability: the extent to which Category 1 (e.g., the emergency services and local authorities) and Category 2 (e.g., public utility companies) organizations can work together effectively in evolving and complex circumstances.^1^ To explore this key issue, we first outline the multiagency structures that are central to an understanding of the UK's response to major incidents, before then turning to the literature on interoperability and strategic decision‐making during emergencies. We argue that an empirically driven theoretical model of intergroup relations is needed and therefore advance the utility of the Social Identity Approach (SIA), before presenting an analysis of the group‐level factors that may have (re)shaped the relationships between local responder agencies and the government during the first year of the pandemic.

### 1.1 The UK's multiagency response to major incidents

The Civil Contingencies Act (herein Civil Contingencies Act, 2004) is the major legislative framework underpinning the UK's national response to major incidents.^2^ The CCA places statutory duties on Category 1 and Category 2 responders to make sure that partner agencies work collaboratively and collectively to respond effectively to emergency situations through their involvement in Local Resilience Forums (LRFs). LRFs are defined by police boundary areas throughout England and Wales, and they are mandated by the CCA to be the ‘…principal mechanism for multi‐agency co‐operation’ in emergency situations (Cabinet Office, 2003, p. 11). Correspondingly, there is a requirement that LRFs meet at least once every 6 months so that the forums can act as the vehicle through which a range of CCA‐mandated duties can be fulfilled. According to the Cabinet Office (2003), these duties relate to (a) assessing the risk of an emergency occurring and planning and preparing accordingly (through the development and maintenance of a Community Risk Register), (b) maintaining and updating emergency and business continuity planning arrangements, (c) communicating with the public, (d) promoting business continuity, (e) sharing information and (f) multiagency cooperation.

In response to a major incident, an LRF stands up a Strategic Coordinating Group. The Strategic Coordination Group (SCG) membership is determined by the type and location of an incident. However, it usually comprises representation from the same Category 1 and 2 organizations as the LRF, as well as other invited senior officers who hold specialist expertise that will contribute to the response. The purpose of the SCG is to provide the overall strategic vision and leadership throughout the duration of the emergency. As such, membership is usually restricted to those who hold the most senior positions within a given organization (e.g., the Chief Constable or Deputy Chief Constable usually represents the police on the SCG).^3^

Beyond these arrangements, some localities in the United Kingdom have chosen to make strategic multiagency investments in a Civil Contingencies Unit (CCU). These comprise teams of specialist planners who support the LRF in preparing for local and/or national emergency incidents. CCUs work to support all responder agencies to meet their statutory duties, to develop and maintain multiagency plans for Category 1 responders, to provide multiagency training for the LRF, to provide 24‐h call out capacity should an emergency event occur and to deliver underlying administrative support to the LRF.^4^

Thus, there is a complex array of multiagency groups involved in a response to a major incident, and it is this framework through which local authorities across the United Kingdom responded to Covid‐19.

### 1.2 Strategic decision‐making during emergencies

While there is a recognition that ‘team’ or group‐level processes and relations are crucial to an understanding of interoperability during emergencies (JESIP, 2021; Pollock, 2013; Power, 2018; Wilkinson et al., 2019), existing research exploring strategic decision‐making tends to focus on the *individual* cognitive processes of the decision‐maker (e.g., Alison et al., 2015; Power, 2015; Shortland et al., 2018). For example, in their review of the literature, CREST (2020) identified four primary factors that impact on command decision‐making. First, that people can ‘buckle’ under time pressure, undermining their capacity to undertake ‘rational’ decision‐making (e.g., Alison et al., 2013, 2015). Second, that people can become overwhelmed by too much information or else have too little information to make accurate decisions (e.g., Alison et al., 2015). Third, that risk and uncertainty can lead to decision‐avoidance (e.g., Alison et al., 2015; van den Heuvel et al., 2012). Fourth, that being accountable for decisions can ‘…lead to cognitive load by considering too much information and overwhelming [the] need to protect ourselves’ (CREST, 2020, p. 2; see also: Alison et al., 2010; Waring et al., 2013). Taken together, the implication of this work is that these four factors can lead to ‘decision‐inertia’ (Power & Alison, 2017), whereby commanders ‘redundantly’ oscillate between the negative consequences that might arise from both acting (to potentially save lives) or not acting (to prevent further harm). Accordingly, solutions proposed by the literature include the importance of training to maximize decision‐maker experience in managing limited cognitive resources (CREST, 2020; House et al., 2013).

Consequently, with the emphasis on individual‐level cognitive processes, there is a danger that this body of work underplays the importance of group‐level relations and the impact that these relationships can have on strategic decision‐making during prolonged major incidents. By contrast to the above, Hill, Guest, Pickford, Hopkinson, Towler, et al. (2020a), Hill, Guest, Pickford, Hopkinson, Daszkiewicz, Whitton, Reed, Towler, et al., (2020b), and Hill, Guest, Pickford, Hopkinson, Daszkiewicz, Whitton, Reed, Thomas, et al. (2020c) conducted three Interim Operational Reviews of the UK's pandemic response between April and October 2020. They reported that national government organizations frequently imposed central control in ways that often disrupted or challenged the preparedness undertaken locally (Hill et al., 2021). Accordingly, this suggests that local strategic leaders during the pandemic have operated in a complex intergroup context and that decision‐making in such circumstances cannot be reduced to an analysis of individual cognitive ‘biases’ (c.f., Cronin & Reicher, 2006, 2009).

### 1.3 The importance of (inter)group processes and relations

Similarly, Davidson et al. (2020a, 2020b, 2020c) have recently argued that the SIA is an important theoretical framework for understanding the relations between local responder agencies and government during major incidents. The SIA jointly comprises Social Identity Theory (Tajfel & Turner, 1979) and Self‐Categorization Theory (Turner et al., 1987; Turner, 1982, 1985). Central to the SIA, is the idea that the self‐concept is hierarchical, comparative and context specific. Consequently, you can define yourself in each context in terms of your personal identity (as ‘I’) or at a more inclusive level in terms of a social identity (based on your psychological group memberships, ‘we’). From this perspective, social identity is ‘…a model of one's position within a set of social relations along with the actions that are possible and desirable given that position’ (Neville & Reicher, 2018, p. 12).

Drawing on the SIA, Davidson et al. (2021, 2022) maintain that for LRF interoperability to be effective, the multiple partners must build a shared sense of identity (a sense of ‘we‐ness’) at the superordinate level (i.e., inclusive of all people involved across organizational boundaries). In so doing, this facilitates the emergence of a new identity with shared norms, values and goals and enhances the partners' ability to work together to overcome challenges. Through interviews with UK emergency service personnel, Davidson et al. (2022) demonstrated that LRFs were able to cultivate a shared sense of identity during the pandemic by drawing on strong pre‐existing relationships and by capitalising on instances where partners shared a sense of common fate (i.e., ‘that we're all in this together’). Where these factors were not present, the SCG Chair played a salient role in strategically facilitating shared identity and group working (e.g., by communicating shared goals to all). A further finding was that the effectiveness of horizontal intergroup relations (i.e., between partners within the LRF) was often undermined by ‘vertical’ intergroup relations (i.e., those between LRFs and government agencies; see Davidson et al., 2021).

### 1.4 The present study

As the above discussion demonstrates, the SIA has important implications for an understanding of how strategic decision‐making functions in major incidents, since the analytical focus is on the complex and changing array of intergroup relationships of those involved in an emergency response (e.g., local stakeholders, national response agencies and government), all of whom differ in terms of status and power (Davidson et al., 2020a, 2020b, 2020c; Hogg & Abrams, 1988). Yet extant research and theory does not tend to explore the complex group‐level factors that (re)shape the relationships between different responder agencies and the government, especially during a prolonged pandemic situation.

Correspondingly, we undertook a case study approach with our focus on the experience of the first year of the COVID‐19 pandemic from the perspective of two senior responders both of whom played key roles within their LRF and SCGs. In so doing, we sought to advance the literature in several important ways. First, previous research (e.g., Crego, 1996; van der Haar et al., 2017; Wilkinson et al., 2019) has often utilized training scenarios to explore multiagency decision‐making. While there is the capacity to create compelling and realistic incidents these are usually relatively small scale and cannot compare to ‘real‐life’ emergency management (Power, 2018). Thus, our study focuses on the strategic decision‐making of responders longitudinally and during a prolonged crisis with an evolving and dynamic political and societal context.

Second, we sought to address the gap between research and practice (Power, 2018). Therefore, our approach was one of coproduction from the outset with our interviewees not only being co‐authors of this study but also informing the analysis and providing ongoing assistance with the policy impact and dissemination of this research.

Third, theoretically, we sought to move beyond individualist explanations for strategic decision‐making during emergencies which focusses on cognitive biases and instead we aim to contribute to the understanding of the social psychological factors that can shape the behaviour of responder agencies during a pandemic. Accordingly, there is a particular emphasis on strategic level decision‐making at the local level and how the relationship with national government impacted on local processes and outcomes.

## 2 METHOD

### 2.1 Data collection

The first, second and fifth author undertook 19 interviews, between March 25, 2020 and February 17, 2021, with a Director of a Civil Contingencies Unit (fourth author) and a Chief Fire Officer (third author), who both played key roles within their LRF and SCG. Additionally, three interviews were undertaken with other Chief Fire Officers across this period, all of whom played senior roles in their respective LRF/SCG. This material informed the analysis and equated to 20h, 28 min and 49 s of audio which were subsequently transcribed. In practice, the interviews usually ranged between 30 min to an hour and took place every two‐to‐three weeks using the online platforms Zoom or Microsoft Teams. The interviews took the form of a debriefing session where we explored interviewees' current challenges and decision‐making, what issues they anticipated they may encounter in the future, as well as following up on any of the issues raised in the previous interview. Additionally, we collected a range of secondary data sources, including newspaper articles, research reports, transcripts of government announcements and media press conferences.

### 2.2 Analytic strategy and presentation of data

The first and second authors undertook a form of applied thematic analysis in line with constructivist versions of grounded theory (Charmaz, 2014). Thus, in accordance with Charmaz (2006), we utilized grounded theory as a set of iterative steps that ‘consist of systematic, yet flexible guidelines for collecting and analyzing qualitative data to construct theories “grounded” in the data themselves’ (p. 2). While the specifics of the analytical process are laid out below, in general we followed the broad analytical stages set out by Bernard and Ryan (1998) and Guest et al. (2012). Specifically, (1) we (re)read the data corpus, including the verbatim interview transcripts; (2) we coded the data and sought to use these codes to develop an initial emergent thematic structure; (3) we then reread the data and initial coding to interrogate, consolidate and revise our thematic structure to make sure that we had developed a parsimonious and comprehensive account; and (4) we sought to build theoretical insights directly from the analysis.

Accordingly, we started the analysis by reading the data corpus to arrange the data both chronologically and thematically. Thus, secondary data sources were used to produce detailed timelines of key national events and issues (e.g., government announcements) and through this process we developed and grouped the interviews into three broad chronological phases or stages. While there is a degree of overlap, the first phase includes the period from the advent of national lockdown in England on the 23rd of March 2020 through to the Prime Minister's announcement indicating that restrictions would be gradually eased on May 10. The second phase then covers the period from May 2020 to the first imposition of ‘tiered’ localized restrictions at the beginning of October. The third phase explores the period from October through to when England went into a further national lockdown in December 2020. Having grouped the transcripts chronologically, they were then (re)read and semantically coded to draw out key episodes as exemplars of theoretical relevance. Accordingly, there was a particular emphasis on the horizontal (inter)group relationships within the LRF/SCG and vertical (inter)group relationships between the LRF/SCG and central government agencies.

Through this process, the first and second author developed a form of ‘composite narrative’ (Willis, 2018) where the perspective of both interviewees (the third and fourth authors) were incorporated to produce a combined account of events. The analysis was then ‘sense checked’ by all authors, two of whom (the third and fourth authors) were able to draw on their considerable and ongoing personal and professional experience to interrogate and refine the content. As Willis (2019) outlines, a ‘composite narrative’ approach has two clear advantages: (1) it facilitates an emphasis on preserving the complicated and contextual accounts of our interviewees and (2) the narrative form ‘… can help to build understanding of particular people and groups, in ways that are accessible to non‐academic audiences’ (p. 477). Once an initial analytical structure was created, we met as a research team several times to discuss, refine, and adapt the analysis so that we achieved the best ‘fit’ with the data set. Quotations presented within the following analysis were all taken directly from the interview transcripts and were judged to be the best exemplars of the themes generated.

## 3 ANALYSIS

### 3.1 Phase 1: March 23 to May 10, 2020

#### 3.1.1 SCG leadership

By the time of the first interview on March 25, a Director of Public Health^5^ (DPH) had been appointed as the Chair of the SCG. The decision to appoint a DPH Chair was in line with the preplanned local response to a flu pandemic. Local responders were told by government to use this guidance as the basis of their response to COVID‐19. As one of our stakeholders suggested:

The reason you put those people in place is that they straddle the local authority and health sectors…but they also work in the local authority, and therefore operate as a traditional Category One responder. (Int. 25/03/20)

However, at that time one of the primary decisions involved declaring a major incident. This triggered discussions as to whether the Chair should shift to the police.^6^ There was some resistance to this idea among several of the stakeholders who wanted to declare a major incident but maintain a health‐led approach. This was primarily because they judged a police force‐led approach would be more ‘command and control’ oriented and therefore undermine the more collaborative multiagency approach that was already in place. To resolve the contention, the SCG compromised by developing ‘trigger’ plans for changing the chair depending on the emerging and evolving context (e.g., the relative salience of the maintenance of public order) and a mechanism whereby partners endorsed the chair at the start of each meeting. This was then adapted to be a decision taken at the end of the meeting to reflect the pace of the response and, if change were to occur, to provide an opportunity to brief the new postholder.

#### 3.1.2 Centralized versus localized response

A salient national issue from the announcement of a national lockdown on March 23 was the need to identify and support vulnerable people. This immediately introduced a logistical problem for the SCG and LRF in identifying who these individuals and households were. This was initially defined via the National Health Service's (NHS) database of clinically extremely vulnerable people, but it was apparent to them that this would not be able to identify everyone at the local level. In parallel, in response to the crisis the Information Commissioner's Office (ICO)^7^ relaxed the rules it had previously imposed in relation to data sharing between local partner agencies. This then enabled the local stakeholders in the LRF to improve their interoperability by sharing data in a manner that had not been seen as possible before the pandemic. As one stakeholder explained:

The relaxation of the rules mean that I now have access to data about those people in this county who are shielded and therefore are the most vulnerable from the worst sort of outcomes from fire in their homes. Same as police, worst outcomes from a point of view of crime as an individual. A reversion back to where the rules were will mean that that will get stopped. So, that sharing and that ability to be thoughtful about how we use the plethora of data that we have about our residents could get lost. (Int. 29/04/20)

Having identified vulnerable people in the county, emphasis then shifted to establishing the infrastructure necessary to deliver food parcels to them. The LRF set up a distribution network involving a central hub with eight district centres. Each centre was then free to agree food procurement contracts locally. However, by the 1st of April it had become apparent to the SCG that the government had decided to centralize control of delivering food parcels and that as a result several locally agreed supply contracts had been subsequently lost to this central procurement process, in ways that undermined supply at the local level. Moreover, when the government food parcels subsequently arrived, they contained produce that was unsuitable for distribution to individual households (e.g., perishable items with short shelf life).

Interviewer: Maybe your expectation was it [government supplied food parcels] was going to be, in a sense, long storage stuff?

Interviewee: Yes. And this was the government's offer. So, the government said, if you bid for it, we can give you X number of thousands of portions to make up food parcels. But what we're getting is not tinned meat and half kilo bags of rice. A 20‐kilo bag of rice having to be split down increases the manual handling and the intervention that needs to be put in place. (Int. 01/04/20)

Food distribution was the first of several issues that formed a pattern over the course of the pandemic whereby government centralization and micromanagement of important aspects of the contingency response disempowered the LRF's emerging ability to creatively solve local problems. For example, by April 10, the government was coming under intense media scrutiny for the failure to provide frontline healthcare staff with adequate Personal Protective Equipment (PPE),^8^ with the Health Secretary announcing that a ‘Herculean effort’ is underway to resolve the issue.^9^ Within the LRF, the operational challenge again revolved around procurement. Rather than developing procurement contracts locally, the LRFs were instructed by Government that the Department for Housing, Communities and Local Government (DHCLG) would be issuing PPE directly to LRFs, with associated directives on how it should be distributed to partner agencies.^10^ The SCG subsequently allocated their supplies of PPE in line with this top‐down instruction only to be told within 36 h that the guidance had changed. This placed the SCG in the difficult position of having to potentially recall and redistribute PPE stocks, causing tension between the LRF partners. As one stakeholder explained:

…the goalposts being moved is not a helper. So, when the LRF are told initially, right, NHS are not to be accessing the drop [of PPE stock] from DHCLG, to then be told within 36h, no, actually you need to be providing it to dentists, GPs, etcetera, which are part of the NHS structure, that is a bit of a frustration, to say the least. (Int. 15/04/20)

By April 14, it was also becoming increasingly clear that nationally the admission of care home residents discharged from hospital without being tested for COVID‐19 was causing outbreak clusters. Yet at the same time, deaths in care homes were not being included in the daily death figures, with several charities including Age UK warning that older people were being ‘airbrushed’ out of official statistics.^11^ Accordingly, in response, the Care Quality Commission (CQC)—a government agency responsible for regulating all health and social care services in England—was mobilized by the Government. A mission statement published on April 15^12^ highlighted that the CQC would endeavour to utilize their national infrastructure to tackle the lack of COVID‐19 testing in the adult social care sector. It also made clear that the CQC would launch a regular data collection intervention to ascertain the number of deaths within the care sector and to identify the specific ‘COVID‐19 related pressures—such as shortages of PPE—from services who provide care for people in their own homes’.^13^

This centralized imposition came as a surprise to the SCG, and several stakeholders felt it undermined their partnerships, with one interviewee describing the CQC on  April 29 as the ‘new player in the response arm’. This concern was amplified when the CQC informed the local partners to stop providing information through to the LRF data collection cell and that instead the CQC would act as the conduit for information and access. Thus, rather than scaffolding the local response, this government‐led national intervention created fissures in the local partners' relationships that they had worked hard to build through the LRF. As one stakeholder summarized:

It's [the CQC] obviously carving its area out and is telling people to not comply with local requests… the implication for that is a huge amount of need for local activity for going back and reemphasising local messaging. (Int. 29/04/20)

A third example, from late April onwards was an increasingly prominent issue nationally, Test, Trace and Isolate. Accordingly, the government appointed Baroness Dido Harding on May 7 as the Chair of the £37 billion national but privately run ‘Test and Trace’ programme.^14^ Thus, by the beginning of May a key issue for the SCG was how the national test and trace infrastructure would intersect with their LRF response. This was especially important given that the Prime Minister had announced plans to significantly ease lockdown restrictions in the coming weeks. The Test and Trace system was designed and rolled out nationally but contained elements of local delivery. Accordingly, two large‐scale testing sites had been set up within the jurisdiction of the SCG as part of the national programme. Again, for the SCG and LRF, the issue revolved around centralized control not meeting local need. For instance, the CQC superseded local arrangements for testing by directing all care homes to apply for testing at the national sites using the government website. However, these locations had not been included on the list of test centres available to the public through ‘gov.uk’. This meant that care home staff were instead being offered testing sites that were hundreds of miles away, despite substantial local capacity.

At the same time, the SCG and LRF were told that contact tracing would be devolved to the Local Authorities.^15^ Because of this, the LRF moved to create a specialist tracing operational cell which was led by a local authority. However, the LRF did not know what the contact tracing was supposed to entail. It was therefore extremely difficult for the tracing cell to mobilize the necessary activity and resources. As one stakeholder suggested:

Now, we're not quite sure what that tracing looks like. Is it going to be swabbing face‐to‐face? Is it going to be contact tracing by telephone? Is it going to be interviews? No idea… and we don't know about the level of activity we're going to have to resource locally. (Int. 13/05/20)

### 3.2 Phase 2: May 10 to September 30, 2020

#### 3.2.1 Liberalization and shifts to localized legal accountability

It is apparent that as early as April 22, the SCG and LRF had reached a level of stability, refining their local response. Their strategic intentions and objectives were formalized and agreed and operational subgroups delivering those objectives were operating with far less strategic direction necessary. This continued period of LRF ‘stabilization’ coincided with a reduction in cases and deaths nationally, with the Prime Minister declaring on April 30 that the United Kingdom is ‘past the peak’.^16^ There was then the announcement by the Prime Minister on May 10 that there would be a ‘phased unlocking’ of restrictions throughout June and July (e.g., allowing meeting non‐household members outside, the reopening of retail outlets, pubs, and restaurants). This was shortly followed by newspaper revelations on May 22 that the Prime Minister's Chief Political Advisor had broken lockdown guidelines to drive his family hundreds of miles from London to Durham. In defending the Adviser's actions, government ministers consistently evoked the notion of ‘personal responsibility’ and choice.^17^

Thus, the liberalization of government restrictions, change in messaging towards ‘stay alert’^18^ and the political rhetoric of ‘common sense’ and ‘personal judgement’ arguably formed part of a framework whereby central government was seeking to devolve the responsibility of pandemic response to local structures (e.g., LRFs) and the public (e.g., employers, employees) to avoid blame for any uptick in cases and deaths in a potential second wave.^19^ This shift by government was thus seemingly about the avoidance of legal accountability and liability. One of our stakeholders paraphrased a powerful account of the situation from the perspective of a key decision‐maker within the SCG:

I think it's like, right folks, we [the Government] fixed it. We're going to give it to you [local responders] and then when it all goes wrong, it's not our fault [the Government]. We gave it to you in good order. It's your fault it's all gone wrong. (Int. 27/05/20)

#### 3.2.2 Local outbreak control

From as early as May 10, the Prime Minister repeatedly suggested that England would implement a ‘whack‐a‐mole’ approach to control outbreaks in particular geographical areas, with localized public health measures being the primary response rather than a return to a national lockdown.^20^ The first area to be subject to this was Leicester. On June 28, The *Sunday Times* reported that the government was considering imposing a localized lockdown in Leicester due to rises in infections.^21^ By June 29, this was announced as policy by the Health Secretary,^22^ with the restrictions (including the closure of non‐essential shops) coming into force the following day.^23^ To support this approach the government set up a £9 billion central agency—the Joint Biosecurity Centre (JBC)^24^—with the aim to scaffold the local response with this new largescale national resource.^25^

However, mirroring their earlier experiences with the CQC, by July 8 a key issue for the SCG and LRF was that there was a complete lack of clarity as to how the fledgling JBC would interface with existing response structures and thus what the JBC's precise role and purpose would be. As one stakeholder described:

…We haven't actually seen roles and responsibilities actually come out. So, who's got power, under which power, under what power? I suspect there will be a significant change from what was to what is. (Int. 08/07/20)

This highlighted to our participants a continued experience of disconnect between the central and local level of the response. For instance, a key lesson they established from the experiences of local authorities in Leicester was the need for quick and operationally meaningful testing data to identify hotspots before spread becomes exponential. However, in common with responders in Leicester, our LRF found that national data gathering often did not correspond to what was needed locally. For example, the national testing data fed back into the national intelligence picture, which was not immediately available to partners within the LRF. By comparison, local testing data fed into local modelling and was immediately accessible. Moreover, the national Pillar 2 community testing data recorded a person's generic occupational data (e.g., ‘factory worker’). This form of data was insufficient to enable an effective response and target local public health interventions, since there was a need for specific employment details and work addresses. As one stakeholder explained:

… If you do want to trace somebody, if you're a food processing worker… you could be anywhere within the county. But if you can pin down what the employer is then that allows you very quickly to collate for outbreak purposes. (Int. 08/07/20)

By contrast, the benefits of locally driven response to community outbreaks were becoming increasingly clear. For example, the SCG had developed a bespoke bandings ‘thermometer’. This was also a ‘live’ and dynamic dashboard that worked on a traffic light classification system (i.e., green, yellow and red) and enabled SCG/LRF partners to work to a shared and commonly agreed framework for how to respond swiftly to local outbreaks. The dashboard utilized both quantitative (e.g., case rates) and qualitative data (e.g., the ‘look and feel’ on the ground in communities) and allowed responders to determine what a local outbreak control response should look like based on the latest health data. The specificity of the data meant that the focus could be narrowed to specific premises or geographical areas, meaning that public health measures could be tailored to the specific circumstances and localities. The utility of this local innovation was highlighted by one of our stakeholders:

It's been really helpful, because what we've been able to do [as partner agencies in the LRF] is align our own way of working to this thermometer. So as an example, deciding on what activities we may or may not be doing in an area based on where we're at with a thermometer [in that location]. (Int. 19/08/20)

As one stakeholder made clear, the dashboard was an outcome of their effective local multiagency partnerships and their collaborative leadership approach to outbreak management. This was highlighted in the LRF's response to two different ‘types’ of spikes in COVID‐19 cases that occurred in August. The first example related to community transmission within households in a specific geographical area. Through access to the locally developed dashboard, the LRF and SCG were able to quickly determine that there were clusters of infections occurring within three geographical wards in the region. These clusters predominantly related to infections of South Asian families who often lived in multigenerational households. To suppress the outbreak, the local authority was able to draw on well‐developed relationships with local community and faith leaders to embed these community leaders in the subsequent response (e.g., setting up testing in Mosques). Consequently, messaging for the importance of testing and maintaining social distancing was led by respected community members rather than ‘unfamiliar people in suits’ (Int. 19/08/20).

The second example related to a business premise rather than household transmission. Through social media the LRF became aware that a pub had breached social distancing rules, with hundreds of people attending over a three‐day period. Unlike instances of geographical community spread, it was not immediately obvious from the data that there had been a serious outbreak since people had attended from across the LRF region and beyond. This example exposed a weakness of the national track and trace system since the central programme relied on venues collecting accurate and complete contact details of those attending. Without this information the nationally employed remote call staff did not have any other capacity to locate those who had been to the pub. Given that the pub in question had only gathered details for four people across the three‐day period, the SCG had to mobilize their multiagency communications group. Accordingly, the printed press, social media and other websites were ‘*milked*’ by LRF partners to encourage people who had been to the pub to attend a pop‐up testing site created in the adjoining beer garden. While the pop‐up site attracted over 1000 people across a three‐day period, by comparison the ‘out‐of‐town’ largescale national testing centre lay relatively dormant, utilizing only 11% of its capacity. Therefore, this incident was subsequently fed back into the DHCLG by the LRF as a ‘problem case’ that emphasized the requirement for testing to be embedded within communities.

By September 15 there were increased numbers of infections across the county and the Upper Tier Local Authorities^26^ had set up Incident Management Teams (IMT) to provide specialist oversight of each significant outbreak of cases. Yet the ability of IMTs to respond and suppress infection rates was significantly curtailed due to the continued inadequacy of the national testing infrastructure. Thus, to address this issue the SCG chose to set up a local testing system to run in parallel to the national system so that the LRF would have access to data and thus be able to undertake fine‐grained contact tracing. This involved establishing an independent booking system, testing locations and a workforce at considerable expense to the local authorities and competition from centralized procurement processes. As one stakeholder described, the SCG and LRF were essentially having to bypass the multibillion‐pound national infrastructure because it was not fit for local need. Consequently, they instead sought to empower and fund local ingenuity and entrepreneurialism.

The county council has said we'll swallow the costs, but they are eye watering. They are making a pragmatic decision about cost versus health and they are throwing money at it. We've done extraordinary things. We've shaken every contact tree we can get to try and find somebody with a lab. We're going internationally now to get that lab capacity. But it's madness. It is absolute madness that we are having to spend hundreds of thousands of pounds on tests that should be provided. The foundation of the government's track and trace system. (Int. 15/09/20)

### 3.3 Phase 3: September 30 to December 31, 2020

#### 3.3.1 Political tiers

On September 30, the Prime Minister warned that the United Kingdom was at a ‘critical moment’ with cases, hospitalizations and deaths all increasing.^27^ A day later the BBC reported that a three‐tiered alert level system would be deployed in England.^28^ Tier 1 would be geographical areas with fewer than 100 Covid cases per 100,000 people and the prevailing national ‘baseline’ measures would be applicable in these areas (e.g., social distancing, facemask wearing on public transport, the ‘Rule of Six’). Tier 2 would involve enhanced measures such as a ban on inside gatherings for areas where cases were above 100 per 100,000. Tier 3 would be applied to those areas with ‘significantly higher rates of transmission’, with ‘full lockdowns’ imposed, including closures of shops and other non‐essential businesses.

By October 2, it was already clear to our stakeholders that a Tier approach would be problematic. For instance, the LRF was aware that their geographical footprint was ‘sandwiched’ between two COVID hotspots from both the north and the south which contained major urban conurbations and thus areas where people travelled in and out for work. By October 14, the implementation issues of the Tier system were becoming more acute, with infections in the LRF's region rising. For example, there was no definition of the indicative triggers for a change in Tiers or the geographical boundary areas and how granular they would be (e.g., whether Tier decisions be made at the ward, district, or borough level).

Moreover, a key issue from the LRF's perspective was the degree to which there would be local consultation with regard to Tier decisions and who ultimately had the final decision. As it materialized, the decision for which Tier each of the two Local Authority areas within the LRF's jurisdiction would be placed in was taken at weekly ‘Bronze’ level national meetings that included DPHs and officials from national bodies such as PHE. The meetings were also attended by the Health Protection Board (HPB) which had ‘…a range of reporting arrangements into key elected members and political meetings’.^29^ With the increased prominence of the HPB, political actors were now playing a direct role in the trajectory of operational decision‐making for the first time. This new political dynamic had the potential to undermine key stakeholder relations at a local level.

The SCG had achieved agreement on the stance to seek to move to a common Tier for the whole county to maintain consistency and standardization for both organizational delivery and public information sharing and compliance. For instance, the police were concerned about the consequences of the alert level changing across the county, with officers having to enforce enhanced Tier 2 restrictions in Local Authority ‘A’ and Tier 1 rules in neighbouring Local Authority ‘B’.

During October 2020, it was indicated that one of the local authority areas would be moving to a different Tier to others in the county. The political dynamic, which included lobbying by political representatives, led to a decision being taken outside the SCG's agreed stance, which ultimately had different impacts upon partners in the LRF. Up until this point in time, the relationships between local and national tiers had predominantly been through officer contact but this marked an added dynamic for the SCG to consider, namely the roles political representatives had now been given within response decision‐making. As one stakeholder summarized:

I suppose the new system, the alert levels and the activity of the Health Protection Board, which has got the elected member representative role, this is where the first time that the elected representative role collides with the response. And we've never had this in any response before, where the elected representatives have been so embedded in the decision making… I think we've all learnt a lot from that. (Int. 28/10/20)

Therefore, while the government had been acting independently of local responders throughout the response, this episode was an example of local responders starting to act independently of each other. This was due to the increasing political dimension of operational response decision‐making which created fissures in well‐established local partnerships. However, the key difference from our stakeholders' perspective was that the SCG were able to utilize their strong personal informal and formal relationships to work through their differences at a local level in a way that was often not possible nationally with government.

Interviewer: How did these concerns play themselves out, and in particular, how did they get repaired?

Interviewee: It was personal contact, actually…And not just the formal, but the informal relationships that we have built up. (Int. 28/10/20)

By October 31 all authorities entered Tier 2 restrictions, yet on the same day the Prime Minister announced a new four‐week lockdown to be implemented from November 5. This again highlighted the lack of communication and coordination from government, with local responders finding out about the national restrictions through the media. This was experienced as completely undermining the LRF's public messaging campaign, which focused on the move from Tier 1 to Tier 2 and the associated curbs on indoor socialising. As one stakeholder surmised:

The messaging was really confused, because we locally had done all of our messaging around moving into Tier 2 across the county… And then there's this big government announcement which wipes everything out that we had done locally. (Int. 11/11/20)

On November 21, towards the end of the second national lockdown, the media reported that the government was planning to implement a stricter Tier system in England once the national lockdown ended on December 2.^30^ This meant that by December 2 both Local Authority areas were placed into Tier 3—the highest level of restrictions—again with no prior local consultation. Instead, the JBC informed the outcome, with no input locally. As one stakeholder put it:

We don't know the names or the faces or the roles of those people that are actually putting those recommendations in. So, that opaque [Tier] system is even worse than it was before. (Int. 02/12/20)

By this point, there had already been considerable speculation in the media about the potential relaxation of restrictions during the Christmas holiday.^31^ Thus, it was extremely difficult for the LRF to plan and communicate with the public effectively, with the regulations changing from Tier 1 to Tier 2, to a national lockdown, to a revamped Tier 3, to the prospect of a Christmas ‘grace’ period in the space of a few weeks.

#### 3.3.2 Concurrency and local resilience

Yet, despite this continuing uncertainty at a national policy level, the LRF and SCG were increasingly confident in their local preparedness to deal with concurrent incidents because of the relationships they had built up throughout the pandemic. Correspondingly, by December 2 their approach was characterized by pragmatism and the reality that other major incidents (including, at this time the prospect of a ‘no deal’ Brexit) would involve the same personnel and so it was inefficient to duplicate response structures. As one stakeholder described:

So, at the moment I'm working on a plan for how we manage the third, fourth and fifth [major incident] at the same time, which isn't unlikely, bearing in mind the weather and going into winter…we will put a pragmatic command and control structure in place which utilises all of these sub‐groups that are existing [for Covid‐19]. And we will ask them to broaden their scope to embrace any impact upon their remit. (Int. 02/12/20)

Thus, the need to administer the vaccine in mid‐December was not viewed by our stakeholders as a new phase of the pandemic but rather a continuation of a long‐running response process, characterized by the disconnect between the national and local level. For instance, the SCG and LRF were initially informed by government that largescale vaccination centres would be set up by the military as part of a national roll‐out plan. However, the local level was then subsequently tasked with building the required infrastructure. Accordingly, this was viewed as simply the latest example of initial centralized government control, followed by decentralization to the local level once delivery problems emerged.

[There has been]…this pattern of behaviour that we've seen from the government, that is: put a national system in place, the national system fails, they scrabble around for a few months, and then they give it to the local level to manage when it's got too hot to handle. Now that isn't a bad thing because we can manage it locally and actually xShire's been really brave. (Int. 02/10/20)

## 4 DISCUSSION

The aim of this paper was to explore the complex group‐level factors that (re)shaped the relationships between different local responder agencies and the government, during the first year of the COVID‐19 pandemic. To achieve this, we undertook a case study approach. From March 2020 to February 2021, we regularly interviewed two senior responders, both of whom played key roles within their LRF and SCG. Through these ethnographic semi‐structured interviews, we documented contemporaneous evidence of the key challenges that they faced, how they overcame these challenges and the issues that they foresaw emerging in the future.

Our analysis suggested that the LRF and SCG developed an effective localized approach toward outbreak control and a growing resilience in dealing with concurrent emergency incidents. Part of this success seemed to revolve around their capacity to build a shared sense of identity between responders at the local level and this togetherness and collaborative leadership empowered their local response (Davidson et al., 2021, 2022; Haslam et al., 2020). Yet all the way through national government agencies imposed central control on aspects of the response in ways that undermined, duplicated, or misaligned with their preparedness locally (see also Hall et al., 2021). Therefore, our analysis cautions against an approach that seeks to artificially untangle ‘horizontal' relationships from ‘vertical’ relationships because the experience and decision‐making of local responders during the pandemic could not be adequately understood without reference to the actions and decisions of other groups such as the government.

Our findings have implications for the theoretical understanding of strategic decision‐making during emergencies. For instance, our case study has challenged the notion of ‘decision‐inertia’ and the corresponding conceptual focus on the ‘ultimate’ decision‐maker (e.g., Alison et al., 2015; Power, 2015, 2018; Shortland et al., 2018), at the expense of studying the complex (inter)group processes and relations that characterize emergency decision‐making and outcomes (see Davidson et al., 2020b, 2020c, 2021, 2022; Stott et al., 2021). For example, our analysis has demonstrated the overt centralized political control of key aspects of the pandemic response, infrastructure, and resourcing that structurally constrained and sometimes actively undermined the capacity of local strategic leaders to respond optimally. Thus, political choices, including the imposition of centralized bodies such as the CQC and JBC, negatively impacted on the outcomes that local leaders were able to deliver in ways that are not explained by those in the LRF and SCG redundantly oscillating between action and inaction (c.f., Power & Alison, 2017). Instead, strategic level local responders were operating and navigating a complex array of intergroup relationships characterized by a continued disconnect between central government and the LRF/SCG (Hill et al., 2021). The ability for the LRF to function successfully in this context often meant that senior leaders had to skilfully navigate central response structures and agencies to empower local partner activity and entrepreneurialism.

Moreover, our analysis highlights the need to broaden the conceptualization of accountability beyond the idea that *personal* responsibility for decision‐making motivates leaders to prioritize their own self‐protection from censure (Alison et al., 2010; CREST, 2020; Waring et al., 2013). Instead, our paper shows that accountability can also operate at the intergroup level – that powerful groups such as a government can operate strategically in ways that seek to offload *collective* or group‐level responsibility to less powerful groups or individuals (Cronin & Reicher, 2006, 2009; Reicher, 2021). Therefore, accountability relates to relations between groups as much as it refers to how leaders handle personal responsibility under intense pressure. Accordingly, strategic decision‐makers must consider and resolve (often competing) accountability concerns from a range of different ‘audiences’ (c.f., Bottoms & Tankebe, 2012) or groups (e.g., ‘the public’, media, politicians). Our work points to the utility of exploring how senior decision‐makers balance or navigate these different accountability concerns (Cronin & Reicher, 2006, 2009; Leach, 2021).

More generally, our analysis highlights the value of a locally embedded and funded public health response to pandemics (Scally et al., 2020). For instance, the analysis showed that the LRF partners were able to utilize their pre‐existing knowledge of their communities and relationships with key community leaders in ways that empowered local people to achieve key goals such as increased testing and enhanced public health messaging in virus ‘hotspots’. Our findings also demonstrated the relative impotence of national ‘remote’ systems such as ‘Track and Trace’ compared to responses that were embedded within communities. Thus, our analysis suggests that local authorities are well placed to garner public support and legitimacy for, and compliance with, public health directives. This is, in part, through their ability to position themselves as ‘*of*’ the community and acting *for* the community (c.f., Radburn & Stott, 2019; Radburn et al., 2018, 2021; Reicher & Stott, 2020; Stott et al., 2020). What is needed is appropriate funding and scaffolding from government agencies to support these activities (Reicher & Stott, 2020).

Yet our case study additionally highlights that the UK government appears to have consistently undermined the principle of subsidiarity throughout the pandemic (c.f., Hill, Guest, Pickford, Hopkinson, Towler, et al., 2020a; Hill, Guest, Pickford, Hopkinson, Daszkiewicz, Whitton, Reed, Towler, et al., 2020b; Hill, Guest, Pickford, Hopkinson, Daszkiewicz, Whitton, Reed, Thomas, et al., 2020c), despite this being a key principle underpinning the Civil Contingencies Act (CCA). Thus, our analysis demonstrates the need to review the government's relationship to operational incident management to protect local authorities from unnecessary central interference. Much like the effective local response to outbreaks relied on the LRF empowering the public, subsidiarity should mean that central authorities scaffold local authorities by providing support for aspects of the response that cannot be delivered locally. Although rare, our analysis did highlight one example of effective centralized scaffolding. The ICO's decision to relax data sharing rules served to empower local interoperability and thus enabled enhanced protection of vulnerable people. Therefore, one implication of our analysis is that there should be greater flexibility for interagency data‐sharing (Waring et al., 2018) where the Civil Contingencies Act may ‘trump’ data protection issues in emergency situations. Accordingly, our work concords with Hill, Guest, Pickford, Hopkinson, Daszkiewicz, Whitton, Reed, Thomas, et al.'s (2020c) recommendation that there should be a centrally commissioned UK government review that scrutinizes the Civil Contingencies Act and how the principle of subsidiarity—that response decision‐making is made at the most local level possible—is properly enacted in future prolonged major incidents.

Theoretically, our findings support the utility of the SIA as an important framework for developing an understanding of the relations between local responder agencies and the government during emergencies. For instance, our analysis points to the salience of strategic leaders at the local level galvanizing a sense of ‘we‐ness’ or superordinate identification within LRFs to enhance collaborative working throughout major incidents. For example, in line with Davidson et al. (2022), the bespoke bandings ‘thermometer’ developed by the SCG appeared to be the outcome of (a) strong pre‐existing relationships between partner organizations and (b) an emergent togetherness developed by partner agencies who shared a sense of common fate (i.e., ‘that we're all in this together’). Moreover, like Davidson et al. (2021), our analysis highlights that the emergence of a shared identity between responders at a local level may also serve as a ‘buffer’ to mitigate some of the negative impacts of dysfunctional relations between local authorities and central governments during major incidents.

Yet despite these insights, there are some important limitations to our analysis that need to be acknowledged. These relate primarily to our case study approach which is both a key strength and weakness. On the one hand, we were able to utilize unfettered and continued access to two senior responders involved in the response of one LRF to the pandemic. This meant that we were able to document and explore the key local issues and challenges as they understood them. Accordingly, we were able to relate their strategic decision‐making at a local level to their ongoing relationship with government at a national level throughout the first year of the response to the pandemic. However, while our approach may have naturalistic generalizability—that is, the findings resonate strongly with those experiences of key responders from other LRFs/SCGs (e.g., Hill et al., 2021), equally, our analysis may be limited to the locality in which we focused on. Additionally, the voices of other key responders within the LRF/SCG we have explored may have changed or challenged the ‘composite narrative’ (Willis, 2018, 2019) we have presented.

Notwithstanding these caveats, our work has contributed to the theoretical understanding of the social psychological factors that can shape the behaviour of responder agencies during a prolonged and unprecedented crisis. Our paper points to the critical importance of the relationships between the groups involved—both in terms of ‘horizontal’ relations within local response structures and the ‘vertical’ relations between the LRF/SCG and government. Our case study analysis demonstrated that during the COVID‐19 pandemic the UK government operated in ways that undermined the principle of subsidiarity and damaged the ways in which the LRF could mobilize to help scaffold local community resilience.

## Data Availability

Research data are not publicly shared.

## ENDNOTES

1 See: https://www.ukfrs.com/sites/default/files/2017-09/JESIP%20Joint%20Doctrine%20‐ %20The%20Interoperability%20Framework. pdf

2 A major incident is defined in the UK by the Joint Emergency Services Interoperability Programme (JESIP, 2021, p. 15) as ‘An event or situation with a range of serious consequences which requires special arrangements to be implemented by one or more emergency responder agency’.

3 In addition to SCGs, it is normative to have a separate Tactical Coordinating Group (TCG). TCGs are ‘…tasked with interpreting strategic direction, developing tactical plans, and coordinating actions and resources’ (Waring et al., 2020, p. 633). Furthermore, there are several Operational thematically organized subgroups that are mobilized to deliver the strategic objectives identified at SCG level (e.g., communications). Thus, a key distinction between the LRF as opposed to the SCG/TCG is that LRFs are engaged in resilience planning and therefore draw on emergency preparedness guidance. Whereas SCGs/TCGs are established to deal with the response to an emergency/incident and are consequently steered by the Cabinet Office's ‘Emergency Response and Recovery’ non‐statutory guidance that sits alongside the CCA See: https://assets.publishing.service.gov.uk/government/uploads/system/uploads/attachment_data/file/253488/Emergency_Response_and_Recovery_5th_edition_October_2013.pdf

4 For example, https://www.dorsetprepared.org.uk/about-the-lrf/the-dorset-civil-contingencies-unit/

5 ‘Directors of public health are responsible for determining the overall vision and objectives for public health in a local area or in a defined area of public health, such as health protection. They are accountable for delivering public health objectives and reporting annually on the outcomes and future work. They may be from any background, but must be qualified specialists in public health and registered with the General Medical Council or General Dental Council or UK Public Health Register’. See https://www.healthcareers.nhs.uk/explore-roles/public-health/roles-public-health/director-public-health

6 Typically, the police will assume the role of the SCG Chairpersonship in an emergency where: ‘There is an immediate threat to human life’; ‘There is a possibility that the emergency was a result of criminal or terrorist activity’; or ‘There are significant public order implications’. See https://www.jesip.org.uk/uploads/media/pdf/5_Strategic_Co-ordinating_Group_Roles_Responsibilities.pdf

7 The ICO is ‘The UK's independent authority set up to uphold information rights in the public interest, promoting openness by public bodies and data privacy for individuals’. See https://ico.org.uk.

8 ‘PPE is equipment that will protect the user against health or safety risks at work. It can include items such as safety helmets, gloves, eye protection, high‐visibility clothing, safety footwear and safety harnesses. It also includes respiratory protective equipment (RPE)’. See https://www.hse.gov.uk/toolbox/ppe.htm

9
https://www.bbc.co.uk/news/uk-52248423

10 It is the SCG rather than the LRF that has a defined response role according to the CCA, indicating a level of government misunderstanding of its own legislation and guidance.

11 See: https://www.bbc.co.uk/news/health-52455072

12
https://www.cqc.org.uk/news/stories/cqc-sets-out-next-steps-support-adult-social-care-during-covid-19-pandemic

13
https://www.cqc.org.uk/news/stories/cqc-sets-out-next-steps-support-adult-social-care-during-covid-19-pandemic

14
https://www.gov.uk/government/news/new-chair-of-coronavirus-test-and-trace-programme-appointed

15 Local authorities are ‘…responsible for a range of vital services for people and businesses in defined areas. Among them are…functions such as social care, schools, housing and planning and waste collection…’. See: https://www.local.gov.uk/about/what-local-government

16 See: https://www.bbc.co.uk/news/uk-52493500

17 E.g. https://www.politicshome.com/news/article/dominic-cummings-row-minister-says-families-should-try-their-best-to-follow-lockdown-rules

18 The ‘roadmap’ towards loosening restrictions on May 10 was accompanied with a change of government messaging from the clear ‘Stay at home, protect the NHS, save lives’ to the near‐universally derided slogan ‘Stay alert, control the virus, save lives’.

19 For instance, a report by the Independent SAGE group of scientists criticized the Prime Minister's speech outlining a ‘return to work’ strategy without providing any framework or guidance as to how that could be safely achieved. See: https://www.independentsage.org/wp-content/uploads/2020/05/The-Independent-SAGE-Report.pdf

20
https://news.sky.com/video/coronavirus-prime-minister-says-well-be-doing-whack-a-mole-on-local-flare-ups-11986745

21 See: https://www.bbc.co.uk/news/uk-england-leicestershire-53206506

22
https://www.bbc.co.uk/news/uk-england-leicestershire-53229371

23
https://www.leicestermercury.co.uk/news/leicester-news/leicester-lockdown-rules-restrictions-confirmed-4279424

24 The JBC was created to fulfil two primary roles: (1) ‘…to provide real‐time analysis about infection outbreaks’ and (2) ‘…to advise on how the government should respond to spikes in infections – for example, by closing schools or workplaces in local areas where infection levels have risen’. See https://www.instituteforgovernment.org.uk/explainers/joint-biosecurity-centre

25
https://www.researchprofessionalnews.com/rr-news-uk-politics-2020-7-government-spending-9bn-on-covid-19-joint-biosecurity-center/

26 ‘In some areas of England, local government is divided between a county council (upper‐tier) and a district council (lower tier), which are responsible for different services. In other areas, there is a single unitary authority instead’. See https://lgiu.org/local-government-facts-and-figures-england/

27
https://www.bbc.co.uk/news/uk-54362900

28
https://www.bbc.co.uk/news/uk-politics-54371943

29
https://www.gov.uk/government/publications/containing-and-managing-local-coronavirus-covid-19-outbreaks/covid-19-contain-framework-a-guide-for-local-decision-makers#roles-and-responsibilities

30
https://www.theguardian.com/world/2020/nov/21/england-to-enter-stronger-three-tier-system-after-lockdown

31
https://www.lbc.co.uk/news/families-allowed-meet-week-christmas-covid-lockdown/
